# 2,4,6-Triphenyl­aniline

**DOI:** 10.1107/S160053681002338X

**Published:** 2010-06-26

**Authors:** Onome Ugono, Stephanie Cowin, Alicia M. Beatty

**Affiliations:** a Department of Chemistry and Biochemistry, Center for Nanoscience, University of Missouri-St. Louis, St. Louis, Missouri, USA

## Abstract

Individual mol­ecules of the title compound, C_24_H_19_N, do not participate in hydrogen-bonding inter­actions due to the steric bulk of the phenyl rings *ortho *to the amine. The dihedral angles between the central ring and the pendant rings are 68.26 (10), 55.28 (10) and 30.61 (11)°.

## Related literature

The reaction of equimolar amounts of pyrazole-3,5-dicarb­oxy­lic acid (HPzDCA) and primary amines have yielded ammonium carboxyl­ate salts that adopt layered architectures, see: Ugono *et al.* (2009 ▶); Beatty *et al.* (2002*a* ▶,*b* ▶). For other amines that do not exhibit inter­molecular hydrogen bonding due to the bulky *ortho* phenyl groups, see: Cherian *et al.* (2005 ▶); Lonkin & Marshal (2004 ▶). For the preparation of 2,4,6-triphenyl­aniline, see: Basu *et al.* (2003 ▶); Paul & Clark (2003 ▶).
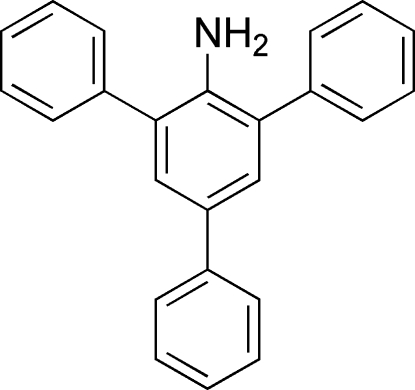


## Experimental

### 

#### Crystal data

C_24_H_19_N
*M* *_r_* = 321.40
Monoclinic, 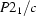

*a* = 10.735 (2) Å
*b* = 14.792 (3) Å
*c* = 11.911 (2) Å
β = 113.02 (3)°
*V* = 1740.7 (6) Å^3^
*Z* = 4
Mo *K*α radiation
μ = 0.07 mm^−1^
*T* = 100 K
0.50 × 0.50 × 0.25 mm

#### Data collection

Bruker SMART APEXII diffractometer
Absorption correction: multi-scan (*SADABS*; Sheldrick, 1996 ▶) *T*_min_ = 0.966, *T*_max_ = 0.983
44061 measured reflections
6695 independent reflections
5813 reflections with *I* > 2σ(*I*)
*R*_int_ = 0.027

#### Refinement

*R*[*F* ^2^ > 2σ(*F* ^2^)] = 0.043
*wR*(*F* ^2^) = 0.125
*S* = 1.02
6695 reflections
226 parameters
H-atom parameters constrained
Δρ_max_ = 0.48 e Å^−3^
Δρ_min_ = −0.23 e Å^−3^

### 

Data collection: *APEX2* (Bruker, 2007 ▶); cell refinement: *SAINT* (Bruker, 2007 ▶); data reduction: *SAINT*; program(s) used to solve structure: *SHELXS97* (Sheldrick, 2008 ▶); program(s) used to refine structure: *SHELXL97* (Sheldrick, 2008 ▶); molecular graphics: *X-SEED* (Barbour, 2001 ▶); software used to prepare material for publication: *SHELXL97* and *PLATON* (Spek, 2009 ▶).

## Supplementary Material

Crystal structure: contains datablocks I, New_Global_Publ_Block. DOI: 10.1107/S160053681002338X/hg2686sup1.cif

Structure factors: contains datablocks I. DOI: 10.1107/S160053681002338X/hg2686Isup2.hkl

Additional supplementary materials:  crystallographic information; 3D view; checkCIF report

## Figures and Tables

**Fig. 1.**
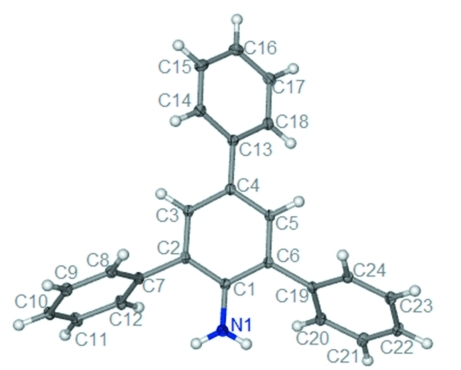
Thermal ellipsoid plot of 2,4,6-triphenylaniline at 50% probability.

## supplementary crystallographic information

### Comment

The reactions of equimolar amounts of pyrazole-3,5-dicarboxylic acid (HPzDCA) and primary amines have yielded ammonium carboxylate salts that adopt layered architectures (Ugono *et al.*, 2009; Beatty *et al.*, 2002a,b). The level of structural fidelity for these organic salts allows, from a crystal engineering point of view, for the tuning of material properties by changing the identity of the organic group for the amines employed in the reaction. The reaction of pyrazole-3,5-dicarboxylic acid and 2,4,6-triphenylaniline (TPA) does not produce appreciable amounts of the desired ammonium carboxylate salt. However, large colorless single crystals of the aniline were obtained and structurally characterized.

The title compound packs in the monoclinic space group *P* 2_1_/*c*, with one molecule in the asymmetric unit. TPA does not self aggregate *via* intermolecular hydrogen bonds in the solid state. This lack of significant intermolecular hydrogen bonds appears to be due to the bulky *ortho* phenyl groups. These groups ensure that the distance requirements for hydrogen bond interactions are not satisfied, as potential participating hydrogen bonding donors and acceptors can not approach each other. This is not uncommon, as other amines, namely 2,6-bis(Benzofuran-2-yl)phenylamine (Lonkin *et al.*, 2004) and (*R*,*R*)-2,6-bis(1-Phenylethyl)4-methylaniline (Cherian *et al.*, 2005) among others, exhibit this characteristic for identical reasons.

### Experimental

Into a 20 ml scintillation vial was placed 65 mg (37 mmol s) of pyrazole-3,5-dicarboxylic acid, 120 mg (37 mmol s) of 2,4,6-triphenylaniline (Basu *et al.*,  2003; Paul & Clark, 2003) and 5 ml of a 3:2 ethanol:water mixture. The mixture was warmed gently until the solution became clear and then filtered. The filtrate was placed in another scintillation vial, and colorless single crystals of the title compound were obtained in 48 h.

### Refinement

All non hydrogen atoms were refined anisotropically. Phenyl hydrogen atoms were placed in calculated positions and treated with a riding model C–H= 0.95 Å, *U*_iso_(H_aryl_)= 1.2*U*_eq_(C) for aromatic carbons. Amine hydrogen atoms were also placed in calulated positions and treated with a riding model N–H= 0.88 Å, *U*_iso_(H_amine_)= 1.2*U*_eq_(N).

### Crystal data

**Table:**

C_24_H_19_N	*F*(000) = 680
*M**_r_* = 321.40	*D*_x_ = 1.226 Mg m^−^^3^
Monoclinic, *P*2_1_/*c*	Melting point = 395–398 K
Hall symbol: -P 2ybc	Mo *K*α radiation, λ = 0.71073 Å
*a* = 10.735 (2) Å	Cell parameters from 6695 reflections
*b* = 14.792 (3) Å	θ = 2.1–33.9°
*c* = 11.911 (2) Å	µ = 0.07 mm^−^^1^
β = 113.02 (3)°	*T* = 100 K
*V* = 1740.7 (6) Å^3^	Prism, colorless
*Z* = 4	0.50 × 0.50 × 0.25 mm

### Data collection

**Table:**

Bruker SMART APEXII diffractometer	6695 independent reflections
Radiation source: fine-focus sealed tube	5813 reflections with *I* > 2σ(*I*)
graphite	*R*_int_ = 0.027
ω scans	θ_max_ = 33.9°, θ_min_ = 2.1°
Absorption correction: multi-scan (*SADABS*; Sheldrick, 1996)	*h* = −16→16
*T*_min_ = 0.966, *T*_max_ = 0.983	*k* = −23→23
44061 measured reflections	*l* = −18→18

### Refinement

**Table:**

Refinement on *F*^2^	Primary atom site location: structure-invariant direct methods
Least-squares matrix: full	Secondary atom site location: difference Fourier map
*R*[*F*^2^ > 2σ(*F*^2^)] = 0.043	Hydrogen site location: inferred from neighbouring sites
*wR*(*F*^2^) = 0.125	H-atom parameters constrained
*S* = 1.02	*w* = 1/[σ^2^(*F*_o_^2^) + (0.0697*P*)^2^ + 0.5511*P*] where *P* = (*F*_o_^2^ + 2*F*_c_^2^)/3
6695 reflections	(Δ/σ)_max_ = 0.001
226 parameters	Δρ_max_ = 0.48 e Å^−^^3^
0 restraints	Δρ_min_ = −0.23 e Å^−^^3^

### Special details

**Table:**

Geometry. All e.s.d.'s (except the e.s.d. in the dihedral angle between two l.s. planes) are estimated using the full covariance matrix. The cell e.s.d.'s are taken into account individually in the estimation of e.s.d.'s in distances, angles and torsion angles; correlations between e.s.d.'s in cell parameters are only used when they are defined by crystal symmetry. An approximate (isotropic) treatment of cell e.s.d.'s is used for estimating e.s.d.'s involving l.s. planes.
Refinement. Refinement of *F*^2^ against ALL reflections. The weighted *R*-factor *wR* and goodness of fit *S* are based on *F*^2^. Conventional *R*-factors *R* are based on *F*, with *F* set to zero for negative *F*^2^. The threshold expression of *F*^2^ > σ(*F*^2^) is used only for calculating *R*-factors (gt) *etc*. and is not relevant to the choice of reflections for refinement. *R*-factors based on *F*^2^ are statistically about twice as large as those based on *F*, and *R*- factors based on ALL data will be even larger.

### Fractional atomic coordinates and isotropic or equivalent isotropic displacement parameters (Å^2^)

**Table:**

	*x*	*y*	*z*	*U*_iso_*/*U*_eq_	
N1	−0.03946 (8)	0.31892 (6)	0.10628 (7)	0.02312 (15)	
H1A	−0.0309	0.3532	0.1693	0.028*	
H1B	−0.1086	0.2817	0.0760	0.028*	
C1	0.05584 (7)	0.32362 (5)	0.05479 (6)	0.01365 (13)	
C2	0.16729 (8)	0.38320 (5)	0.10276 (7)	0.01385 (13)	
C3	0.26324 (8)	0.38694 (5)	0.05077 (7)	0.01493 (13)	
H3	0.3376	0.4272	0.0842	0.018*	
C4	0.25338 (7)	0.33322 (5)	−0.04924 (7)	0.01409 (13)	
C5	0.14616 (7)	0.27160 (5)	−0.09177 (7)	0.01405 (13)	
H5	0.1399	0.2322	−0.1567	0.017*	
C6	0.04796 (7)	0.26577 (5)	−0.04244 (6)	0.01310 (13)	
C7	0.18451 (8)	0.44422 (5)	0.20775 (7)	0.01407 (13)	
C8	0.29191 (8)	0.43079 (5)	0.32071 (7)	0.01645 (14)	
H8	0.3517	0.3812	0.3314	0.020*	
C9	0.31156 (8)	0.48981 (6)	0.41751 (7)	0.01874 (15)	
H9	0.3837	0.4796	0.4941	0.022*	
C10	0.22601 (9)	0.56362 (6)	0.40238 (7)	0.01889 (15)	
H10	0.2404	0.6042	0.4681	0.023*	
C11	0.11933 (9)	0.57760 (6)	0.29049 (7)	0.01905 (15)	
H11	0.0609	0.6280	0.2797	0.023*	
C12	0.09793 (9)	0.51788 (5)	0.19407 (7)	0.01751 (14)	
H12	0.0239	0.5273	0.1184	0.021*	
C13	0.34837 (8)	0.34425 (5)	−0.11131 (7)	0.01444 (13)	
C14	0.48238 (8)	0.37311 (6)	−0.04755 (7)	0.01809 (14)	
H14	0.5146	0.3829	0.0380	0.022*	
C15	0.56863 (8)	0.38749 (6)	−0.10796 (8)	0.02019 (15)	
H15	0.6589	0.4071	−0.0633	0.024*	
C16	0.52351 (9)	0.37333 (6)	−0.23338 (8)	0.02020 (15)	
H16	0.5819	0.3842	−0.2747	0.024*	
C17	0.39142 (9)	0.34296 (6)	−0.29744 (8)	0.01904 (15)	
H17	0.3603	0.3318	−0.3826	0.023*	
C18	0.30489 (8)	0.32897 (5)	−0.23725 (7)	0.01613 (14)	
H18	0.2150	0.3088	−0.2821	0.019*	
C19	−0.06390 (7)	0.19929 (5)	−0.09729 (6)	0.01294 (12)	
C20	−0.19933 (8)	0.22763 (5)	−0.14897 (7)	0.01598 (14)	
H20	−0.2206	0.2894	−0.1433	0.019*	
C21	−0.30318 (8)	0.16641 (5)	−0.20861 (7)	0.01793 (14)	
H21	−0.3944	0.1866	−0.2431	0.022*	
C22	−0.27334 (8)	0.07567 (5)	−0.21765 (7)	0.01761 (14)	
H22	−0.3438	0.0339	−0.2587	0.021*	
C23	−0.13918 (8)	0.04676 (5)	−0.16595 (7)	0.01747 (14)	
H23	−0.1184	−0.0151	−0.1715	0.021*	
C24	−0.03506 (8)	0.10781 (5)	−0.10612 (7)	0.01537 (13)	
H24	0.0559	0.0872	−0.0712	0.018*	

### Atomic displacement parameters (Å^2^)

**Table:**

	*U*^11^	*U*^22^	*U*^33^	*U*^12^	*U*^13^	*U*^23^
N1	0.0255 (3)	0.0285 (4)	0.0204 (3)	−0.0110 (3)	0.0144 (3)	−0.0100 (3)
C1	0.0160 (3)	0.0130 (3)	0.0116 (3)	−0.0006 (2)	0.0051 (2)	0.0004 (2)
C2	0.0169 (3)	0.0120 (3)	0.0117 (3)	−0.0010 (2)	0.0047 (2)	−0.0007 (2)
C3	0.0165 (3)	0.0133 (3)	0.0141 (3)	−0.0019 (2)	0.0050 (2)	−0.0016 (2)
C4	0.0147 (3)	0.0134 (3)	0.0139 (3)	−0.0009 (2)	0.0053 (2)	−0.0010 (2)
C5	0.0153 (3)	0.0124 (3)	0.0139 (3)	−0.0006 (2)	0.0052 (2)	−0.0015 (2)
C6	0.0146 (3)	0.0112 (3)	0.0123 (3)	−0.0006 (2)	0.0040 (2)	−0.0001 (2)
C7	0.0177 (3)	0.0130 (3)	0.0113 (3)	−0.0022 (2)	0.0054 (2)	−0.0006 (2)
C8	0.0166 (3)	0.0180 (3)	0.0134 (3)	−0.0012 (2)	0.0045 (2)	−0.0003 (2)
C9	0.0196 (3)	0.0231 (4)	0.0123 (3)	−0.0046 (3)	0.0049 (3)	−0.0016 (3)
C10	0.0253 (4)	0.0192 (3)	0.0140 (3)	−0.0058 (3)	0.0095 (3)	−0.0039 (3)
C11	0.0268 (4)	0.0151 (3)	0.0165 (3)	0.0002 (3)	0.0098 (3)	−0.0012 (2)
C12	0.0229 (3)	0.0148 (3)	0.0132 (3)	0.0011 (3)	0.0052 (3)	0.0004 (2)
C13	0.0153 (3)	0.0125 (3)	0.0155 (3)	−0.0007 (2)	0.0060 (2)	−0.0011 (2)
C14	0.0154 (3)	0.0195 (3)	0.0182 (3)	−0.0013 (2)	0.0054 (3)	−0.0018 (3)
C15	0.0159 (3)	0.0192 (3)	0.0262 (4)	−0.0002 (3)	0.0090 (3)	−0.0002 (3)
C16	0.0214 (4)	0.0173 (3)	0.0264 (4)	0.0023 (3)	0.0142 (3)	0.0026 (3)
C17	0.0245 (4)	0.0166 (3)	0.0186 (3)	0.0012 (3)	0.0112 (3)	0.0001 (3)
C18	0.0181 (3)	0.0144 (3)	0.0158 (3)	−0.0012 (2)	0.0065 (3)	−0.0017 (2)
C19	0.0153 (3)	0.0117 (3)	0.0117 (3)	−0.0009 (2)	0.0052 (2)	0.0001 (2)
C20	0.0162 (3)	0.0129 (3)	0.0166 (3)	0.0004 (2)	0.0041 (2)	0.0001 (2)
C21	0.0162 (3)	0.0162 (3)	0.0178 (3)	−0.0010 (2)	0.0028 (3)	0.0006 (2)
C22	0.0189 (3)	0.0152 (3)	0.0167 (3)	−0.0041 (2)	0.0048 (3)	−0.0012 (2)
C23	0.0204 (3)	0.0123 (3)	0.0204 (3)	−0.0017 (2)	0.0088 (3)	−0.0018 (2)
C24	0.0168 (3)	0.0124 (3)	0.0176 (3)	−0.0007 (2)	0.0075 (3)	−0.0006 (2)

### Geometric parameters (Å, °)

**Table:**

N1—C1	1.3850 (11)	C12—H12	0.9500
N1—H1A	0.8800	C13—C18	1.4040 (11)
N1—H1B	0.8800	C13—C14	1.4050 (11)
C1—C2	1.4142 (11)	C14—C15	1.3933 (12)
C1—C6	1.4156 (10)	C14—H14	0.9500
C2—C3	1.3951 (11)	C15—C16	1.3944 (13)
C2—C7	1.4939 (11)	C15—H15	0.9500
C3—C4	1.4010 (11)	C16—C17	1.3960 (13)
C3—H3	0.9500	C16—H16	0.9500
C4—C5	1.3987 (10)	C17—C18	1.3930 (12)
C4—C13	1.4841 (11)	C17—H17	0.9500
C5—C6	1.3959 (11)	C18—H18	0.9500
C5—H5	0.9500	C19—C24	1.4012 (11)
C6—C19	1.4907 (10)	C19—C20	1.4030 (11)
C7—C12	1.3997 (11)	C20—C21	1.3958 (11)
C7—C8	1.4020 (12)	C20—H20	0.9500
C8—C9	1.3950 (11)	C21—C22	1.3939 (12)
C8—H8	0.9500	C21—H21	0.9500
C9—C10	1.3923 (13)	C22—C23	1.3938 (12)
C9—H9	0.9500	C22—H22	0.9500
C10—C11	1.3916 (13)	C23—C24	1.3962 (11)
C10—H10	0.9500	C23—H23	0.9500
C11—C12	1.3949 (11)	C24—H24	0.9500
C11—H11	0.9500		
			
C1—N1—H1A	120.0	C7—C12—H12	119.7
C1—N1—H1B	120.0	C18—C13—C14	117.95 (8)
H1A—N1—H1B	120.0	C18—C13—C4	120.56 (7)
N1—C1—C2	120.47 (7)	C14—C13—C4	121.46 (7)
N1—C1—C6	120.92 (7)	C15—C14—C13	120.93 (8)
C2—C1—C6	118.53 (7)	C15—C14—H14	119.5
C3—C2—C1	120.01 (7)	C13—C14—H14	119.5
C3—C2—C7	118.45 (7)	C14—C15—C16	120.51 (8)
C1—C2—C7	121.53 (7)	C14—C15—H15	119.7
C2—C3—C4	122.11 (7)	C16—C15—H15	119.7
C2—C3—H3	118.9	C15—C16—C17	119.14 (8)
C4—C3—H3	118.9	C15—C16—H16	120.4
C5—C4—C3	117.09 (7)	C17—C16—H16	120.4
C5—C4—C13	121.31 (7)	C18—C17—C16	120.38 (8)
C3—C4—C13	121.54 (7)	C18—C17—H17	119.8
C6—C5—C4	122.53 (7)	C16—C17—H17	119.8
C6—C5—H5	118.7	C17—C18—C13	121.06 (8)
C4—C5—H5	118.7	C17—C18—H18	119.5
C5—C6—C1	119.59 (7)	C13—C18—H18	119.5
C5—C6—C19	117.89 (6)	C24—C19—C20	118.49 (7)
C1—C6—C19	122.51 (7)	C24—C19—C6	120.41 (7)
C12—C7—C8	118.77 (7)	C20—C19—C6	120.94 (7)
C12—C7—C2	120.91 (7)	C21—C20—C19	120.92 (7)
C8—C7—C2	120.26 (7)	C21—C20—H20	119.5
C9—C8—C7	120.42 (8)	C19—C20—H20	119.5
C9—C8—H8	119.8	C22—C21—C20	120.14 (7)
C7—C8—H8	119.8	C22—C21—H21	119.9
C10—C9—C8	120.33 (8)	C20—C21—H21	119.9
C10—C9—H9	119.8	C23—C22—C21	119.36 (7)
C8—C9—H9	119.8	C23—C22—H22	120.3
C11—C10—C9	119.64 (7)	C21—C22—H22	120.3
C11—C10—H10	120.2	C22—C23—C24	120.65 (7)
C9—C10—H10	120.2	C22—C23—H23	119.7
C10—C11—C12	120.22 (8)	C24—C23—H23	119.7
C10—C11—H11	119.9	C23—C24—C19	120.44 (7)
C12—C11—H11	119.9	C23—C24—H24	119.8
C11—C12—C7	120.61 (8)	C19—C24—H24	119.8
C11—C12—H12	119.7		

## References

[bb1] Barbour, L. J. (2001). *J. Supramol. Chem.* **1**, 189–191.

[bb2] Basu, B., Das, P., Bhuiyan, M. M. H. & Jha, S. (2003). *Tetrahedron Lett.* **44**, 3817–3820.

[bb3] Beatty, A. M., Grange, K. E. & Simpson, S. E. (2002*a*). *Chem. Eur. J.* **8**, 3254–3259.

[bb4] Beatty, A. M., Schneider, C. L., Simpson, A. E. & Zaher, J. L. (2002*b*). *CrystEngComm*, **4**, 282–287.

[bb5] Bruker (2007). *APEX2 *and**SAINT** Bruker AXS Inc., Madison, Wisconsin, USA.

[bb6] Cherian, A. E., Domski, G. J., Rose, J. M., Lobkovsky, E. B. & Coates, G. W. (2005). *Org. Lett.* **7**, 5135–5137. 10.1021/ol051916j 16268521

[bb7] Lonkin, A. S. & Marshal, W. J. (2004). *Organometallics*, **23**, 3276–3283.

[bb8] Paul, S. & Clark, J. H. (2003). *Green Chem.* **5**, 635–638.

[bb9] Sheldrick, G. M. (1996). *SADABS* University of Göttingen, Germany.

[bb10] Sheldrick, G. M. (2008). *Acta Cryst.* A**64**, 112–122. 10.1107/S0108767307043930 18156677

[bb11] Spek, A. L. (2009). *Acta Cryst.* D**65**, 148–155. 10.1107/S090744490804362X PMC2631630 19171970

[bb12] Ugono, O., Rath, N. P. & Beatty, A. M. (2009). *Cryst. Growth Des.* **9**, 4595–4598.

